# How Do Specific Social Supports (Family, Friend, and Specialist) Reduce Stress in Patients With Substance Use Disorders: A Multiple Mediation Analysis

**DOI:** 10.3389/fpsyt.2021.618576

**Published:** 2021-07-09

**Authors:** Chunyu Yang, Mengfan Xia, Tianshu Li, You Zhou

**Affiliations:** ^1^Nanjing University of Information Science and Technology, Research Institute of Climatic and Environmental Governance, Institute of Prison Sociology and Anthropology, College of Law and Political Science, Nanjing, China; ^2^Department of Sociology, Nanjing University, Nanjing, China; ^3^Faculty of Arts and Social Science, The University of Sydney, Sydney, NSW, Australia; ^4^The University of Melbourne, Parkville, VIC, Australia

**Keywords:** perceived social support, resilience, affect balance, stress, patients with SUD

## Abstract

Perceived social support has been found to reduce the stress of individuals who suffer from substance use disorders. However, the mediating effects of resilience and affect balance in the relationships between specific social supports (family, friend, and significant others) and perceived stress are still unclear. This study focused on substance use disorders (SUD) patients, exploring the mediating roles of resilience and affect balance on the relationships between three dimensions of social supports (family, friend, and specialist) and stress. Three hundred thirty-nine participants completed questionnaires of perceived social support, resilience, affect balance, and stress. After controlling resilience and affect balance, the results suggested the effects of perceived family and specialist supports on perceived stress were fully mediated, and the association between perceived friend support and perceived stress is partially mediated. The multiple mediation analysis showed resilience is significant in mediating the relationship between specific perceived supports in all models, while affect balance is only significant in mediating the relationship between specialist support and perceived stress. Implications for enriching current theoretical research and strategies for government and practitioners were also discussed.

## Introduction

Substance abuse disorder is a worldwide public health problem. It has brought many social stressors on patients with SUD, such as unemployment, stigmatization, and discrimination (1), forming a vicious circle of social isolation, which in turn increases the risk of relapse (2). In addition, stress may overwhelmingly lead to individuals' physical and psychological disorders, such as depression (3), cognitive function impairment (4), cardiovascular disease, and even mortality (5). Therefore, it is theoretically and practically essential to explore the possible methods of reducing stress levels in individuals who abuse substances.

### Perceived Stress and Perceived Social Support

Perceived stress refers to the perceived contradictions that external and internal demands exceed individuals' adaptability (6). Stress is not an inherent issue. Instead, it depends on whether individuals have sufficient capacity and social networks to cope with. Numerous studies have denoted that perceived social support is beneficial for health-promoting behaviors that protect individuals from the negative effects of stressful life events (7, 8). Perceived social support is conceptualized as available resources and assistance given by accessible individuals or groups that may help individuals address stressful events (9).

The cognitive-transactional stress theory (10) denotes that perceived stress is a dynamic cognitive assessment process, including potential stress events, primary cognitive assessment of events, secondary assessments, coping style choices, and outcomes of adaptation. Individuals' assessment and cognition of stress determine the choice of individual coping styles. An individual's ability to address stress depends on the individual's belief that there are sufficient resources and social support to counteract the harmful impacts of stress. People with high social support tend to experience better mind and body conditions, which are conducive to enhancing self-confidence, providing supports for reducing personal stress (11). Empirical studies show that perceived social support helps individuals overcome the adverse impacts of stressful events that improve their social adaptability (12). Given dimensions of social support (i.e., relationship-specific support) are distinct constructs, it is recommended to study the roles of three types of social supports (family, friend, and specialist supports) (13). From empirical perspectives, unmasking the underlying relationship between different dimensions of social support and stress in patients with SUD has enormous significance in updating the knowledge and measurements regarding designing specific treatments and interventions to reduce SUD individuals' stress and promote their integral well-beings. Therefore, the current study was designed for investigating the potential mediation roles of resilience and affect balance between three subtypes of perceived social support and stress in individuals with SUD. Based on the theoretical and empirical literature, it is expected to propose the first hypothesis of the current study:

*H1. Specific perceived social supports (family, friend, and specialist) have a significant and negative direct effect on perceived stress;*

#### Perceived Social Support, Resilience, Affect Balance, and Stress

Resilience is defined as a trait that is beneficial for buffering the detrimental effects of stressors and adversities and promoting positive bio-psycho-social functions (14). Resilience is a resource and asset of an individual, which can help deal with adverse conditions and make rational choices, generating positive outcomes (15). Recently, an increasing number of scholars have been paying attention to the protective factors embedded in individuals' social environment, aiming to help individuals utilize various active resources to promote ideal results (16). Family, peer groups, and important others are crucial social capital for individual development, especially drug users. Wang et al. (17) suggest that social support is an essential external protective factor that can promote resilience. Drug users with sufficient social networks can adjust themselves more effectively and efficiently when facing adversity and difficulties (7, 18). Also, Cai et al. (19) has suggested that resilience is a mediator between perceived social support and perceived stress. Individuals owning high levels of perceived social support tend to obtain assistance from their social network, which transforms the social capital into psychological capital, enhancing the ability to bounce back while confronting stressful events (20). Based on the antecedent literature, it is rational to propose the second hypothesis of the current study:

*H2. Resilience mediates the associations between specific perceived social supports (family, friend, and specialist support) and stress;*

Affect balance may be another potential mediator of the association between specific perceived social supports and perceived stress. Affect balance denotes the capability of balancing positive and negative affections. People with higher scores of affect balance have more positive affect than negative affect (21). Studies have identified perceived social support is a significant predictor of affect balance (22). For example, prior research suggests that perceived social support is positively correlated with affect balance and self-esteem (23) and negatively correlated with loneliness among SUD patients (24). Further, some research suggests that affect balance is negatively associated with stress (25, 26). Based on the broaden-and-build theory (27), positive emotions can help individuals address challenges with a positive attitude, broaden their thinking, expand their perceptions, and choose more creative and flexible actions, while negative emotions often narrow individuals' abilities. The theory holds that affect balance can activate actions, expand awareness, buildings resources, and relieve stress (27). Stroud et al. (28) outlined a physiological model that affective states are significantly associated with individuals' health and well-being. Poor affect balance is closely linked with a high level of perceived stress. In a word, individuals with higher perceived social support tend to make better use of the surrounding environment and, in turn, enhance their performance of affect balance, helping them to cope with stress (29). Given the findings from theoretical and empirical studies, it is expected to propose the third hypothesis:

*H3. Affect balance mediates the associations between specific perceived social supports (family, friend, and specialist support) and perceived stress;*

In addition, it should be noted that resilience and affect balance are not independent mediators. Substantial studies have suggested that resilience can promote affect balance (30–32). It has been shown that individuals with a higher level of resilience can deal with their emotional conflicts more successfully (33–35). Therefore, it is expected to assume that perceived social support impacted stress through serial-mediation effects of resilience and affect balance. Therefore, we proposed the fourth hypothesis regarding serial mediations:

*H4. The serial mediation path of perceived social support (family, friend, and specialist support)*→*resilience*→*affect balance*→*perceived stress is significant;*

#### The Current Study

**Figure 1:**
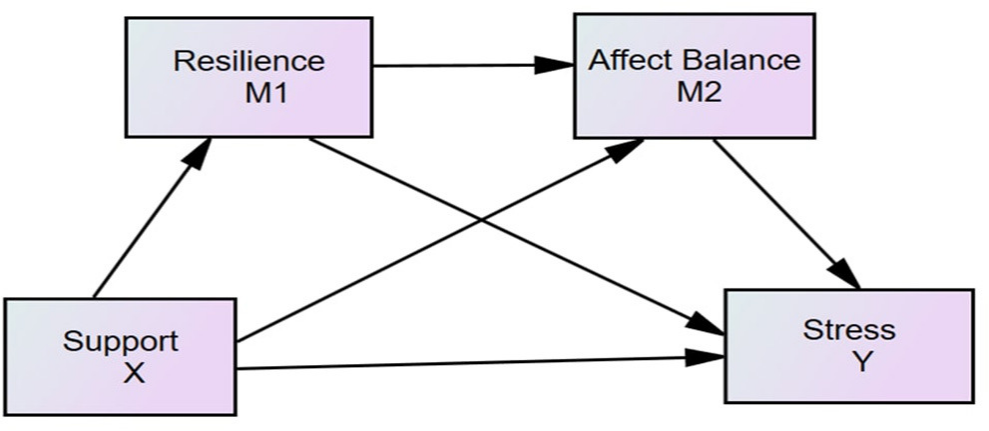
Hypothesized model. X, independent variable; Y, dependent variable; M1, the first mediator; M2, the second mediator; Support, family/friend/specialist/supports; Stress, perceived stress.

To our knowledge, although there are numerous studies on the relationship between specific perceived social supports and stress, there is no research that simultaneously investigates the relationships between specific social supports and stress, especially among patients with SUD. Based on the antecedent literature, the current study proposed three models, examining the effects of specific perceived social supports (family, friend, and specialist, respectively), on perceived stress in individuals diagnosed with severe SUD addiction level. The hypothesized model is shown in Figure 1.

## Method

### Participants and Procedure

All patients (256 males and 83 females) who volunteered to participate in the study were from two compulsory rehabilitation centers in a southern city of China. They were informed of the detailed information of the research project and confidentiality before joining the project. The whole survey took about 30 min, during which participants were allocated separately in independent conference rooms. All 339 patients were from 20 to 60 years old [mean age = 38.64 years, standard deviation (S.D.) = 9.10 years]. 69.3% of the participants were relapsers in the sample, and 30.7% were getting their first treatment. For the drug consumption history, 18.4% of the participants used drugs <5 years, 30.2% of the participants took drugs from 5 to 10 years, and 51.4% took drugs more than 10 years. For the last time of taking illicit drugs, 17.4% of the participants were 1 month ago, 23.1% of the participants were 1–3 months ago, 16.5% of the participants were 3–6 months ago, and 42.5% of the participants were more than half a year ago. The detailed demographic information is shown in Table 1.

**Table 1:** Sample characteristics.

**Sample characteristics**		**Total (*****N*** **=** **339)**		**Male**		**Female**	
		**M**	**SD**	***n***	**%**	***n***	**M**
Age (20–60 years)		38.64	9.10	–	–	–	–
		***n***	**%**				
Gender	1. Male	256	75.5	–	–	–	–
	2. Female	83	24.5	–	–	–	–
Education (*n* = 332)	1. Elementary school and below	61	18.0	50	19.5	11	13.3
	2. Middle school	181	53.4	132	51.6	49	59.0
	3. High school	68	20.1	54	21.1	14	16.9
	4. College and above	22	6.5	13	5.1	9	10.8
Marital status	1. Single	107	31.6	83	32.4	24	28.9
	2. Married	115	33.9	83	32.4	32	38.6
	3. Divorced	109	32.2	84	32.8	25	30.1
	4. Widowed	8	2.4	6	2.3	2	24
Annually Income (yuan/year) (*n* = 329)	<10,000	95	28.0	59	23.0	36	43.4
	10,000–50,000	112	33.0	82	32.0	30	36.1
	50,000–100,000	66	19.5	56	21.9	10	12.0
	100,000–200,000	32	9.4	26	10.2	6	7.2
	>200,000	24	7.1	24	9.4	0	0
Work status (*n* = 338)	1. Unemployment	203	59.9	152	59.4	51	61.4
	2. Famer	16	4.7	12	4.7	4	4.8
	3. Worker	6	1.8	5	2.0	1	1.2
	4. Individual business	60	17.7	52	20.3	8	9.6
	5. Servicer	12	3.5	7	2.7	5	6.0
	6. Company stuff	16	4.7	12	4.7	4	4.8
	7. Government stuff	1	0.3	1	0.4	0	0
	8. Others	24	7.1	15	5.9	9	10.8
Substance classification	1. Heroin	97	28.6	73	28.5	24	21.7
	2. Methamphetamine	222	65.5	169	66.0	53	63.9
	3. Marihuana	7	2.1	5	2.0	2	2.4
	4. Ketamine	2	0.6	2	0.8	0	0
	5. Morphine	3	0.9	3	1.2	0	0
	6. MDMA (ecstasy)	2	0.6	0	0	2	2.4
	7. Others	6	1.8	3	1.2	3	3.6

### Measures

#### Perceived Social Support

Multi-dimensional Scale of Perceived Social Support (MSPSS) was implemented to measure perceived social support. MSPSS has three subscales: perceived family support subscale, perceived friends support subscale, and perceived specialists support subscale. Participants were requested to rate this 7-point scale from 1 = “strongly disagree” to 7 = “strongly agree” (36). The scores of specific perceived social supports were added up by all items in every subscales. A higher score represents a higher level of specific perceived social support. The scale of MSPSS has shown satisfactory consistency and is widely applied among Chinese groups (37). In the present study, Cronbach's alpha of the perceived family, friend, specialist supports were 0.858, 0.825, 0.821, which suggests that all subscales of perceived social supports have good reliability.

#### Resilience

The Connor-Davidson Resilience Scale (CD-RISC) has 25 items used to measure the ability to deal with adversity. Participants were asked to rate on CD-RISC based on their perception of each item over the last month (38). The scale consists of items such as “I can adapt to change,” “I have a close and safe relationship,” and “No matter what happens, I can handle it.” CD-RISC is a four-point Likert scale (0 = “none at all,” 4 = “almost always”), with a total score from 0 to 100. Higher scores represent greater resilience. Previous studies suggest that the Chinese version of the CD-RISC indicated excellent reliability (39, 40). The Cronbach's alpha of CD-RISC was 0.900, which suggests that CD-RISD has very good reliability in the current study.

#### Affect Balance

Affect balance was assessed with the Positive and Negative Affect Scale (PANAS) designed by Watson et al. (41). The questionnaire consists of 20 items, including two subscales of positive affect (10 positive affections, e.g., “interested” and “enthusiastic”) and negative affect (10 negative affections, e.g., “guilty” and “distressed”). PANAS is a 5-points scale evaluating individuals' positive and negative feelings over last few weeks (1 = very slight or not at all, 2 = relatively small, 3 = moderate, 4 = considerable, 5 = very strong). The score was calculated by subtracting the sum of negative affect items from the sum of positive affect items (42). Previous studies have shown that the PANAS in Chinese indicates good reliability and validity (24). The Cronbach's alpha of PANAS was 0.890, indicating good reliability in the current study.

#### Perceived Stress

This study applied the Perceived Stress Scale (PSS) for measuring the perceived pressure of the patients. The PSS is a 14-item scale in which participants can rate their perceptions regarding how much stress they have experienced over the last 4 weeks (43). PSS has four points 4-points scale, with response value ranging from 0 (never) to 4 (frequent). A higher score reflects a higher level of perceived stress. The PSS-14 scale has revealed high reliability in the Chinese context (44). Cronbach's alpha of PSS was 0.706, indicating acceptable reliability. The values of Cronbach's alpha of all measured scales indicated at least the acceptable reliability standard for the following mediation analysis.

#### Addiction Severity Diagnostic Questionnaire (DSM-5)

This study implemented a DSM-5-based self-reported questionnaire to measure the addiction severity of the participants. The DSM-5 addiction severity is measured by evaluating 11 symptoms/criteria on the subjects (45). The symptoms include (1) craving; (2) tolerance; (3) hazardous use; (4) withdrawal; (5) prolonged use of substantial amounts; (6) collapse of relational and social connections; (7) withdrawal from social and occupational events; (8) use-related physical and psychological issues; (9) substantial using time; (10) social and interpersonal problems related to use; (11) repeated attempts of abstinence (45). There are three levels of addiction severity which are measured by counting the symptoms/criteria: 2 to 3 is mild level, 4 to 5 is moderate level, 6 and above is severe level (45). The results showed that all 339 participants were diagnosed with severe addiction. The primary reason was that they accepted abstinence treatment in a compulsory rehabilitation center, which is a mandatory drug treatment center specializing in curing patients with chronic SUD. In this study, its Cronbach's alpha was 0.730.

#### Data Analyses

In this study, IBM SPSS version 22 was utilized to analyze the descriptive statistics. Pearson analysis was operated to examine the bivariate correlation between social supports (family, friend, and specialist supports), resilience, affect balance, and stress. Then, we created serial multiple mediation models to investigate the mediation effects of resilience and affect balance on the association between family, friend, specialist social supports, and perceived stress. The bootstrapping in SPSS PROCESS macro was used to test the mediation effects of the study (46). By random sampling, 10,000 samples were generated and employed the 95% confidence interval (CI) in the analysis of the mediation effects (47). If lower and upper bounds of 95% CI do not span zero, the path is significant at the 0.05 level. The analyses were administered for all models.

## Results

### Preliminary Analyses

Overall, we calculated the means, standard deviations (S.D.), Cronbach coefficients, and bivariate correlations (see Table 2). The results suggested all variables were significantly correlated.

**Table 2:** Means, standard deviations (SD), reliabilities and inter-correlations among study variables.

**Number**	**Measure**	**Mean**	**SD**	**Alpha**	**1**	**2**	**3**	**4**	**5**	**6**
1	Family support	18.45	5.45	0.858	1					
2	Friend support	16.52	5.13	0.825	0.495^**^	1				
3	Specialist support	17.35	5.56	0.821	0.745^**^	0.570^**^	1			
4	Perceived social support	52.33	14.08	0.906	0.874^**^	0.796^**^	0.912^**^	1		
5	Resilience	76.67	16.64	0.900	0.402^**^	0.426^**^	0.502^**^	0.523^**^	1	
6	Affect balance	1.70	7.15	0.890	0.221^**^	0.201^**^	0.291^**^	0.278^**^	0.300^**^	1
7	Stress	41.20	5.64	0.706	−0.130^**^	−0.278^**^	−0.282^**^	−0.231^**^	−0.386^**^	−0.313^**^

*α = Cronbach's alpha*.

** *significant at the 0.01 level (2-tailed)*.

#### Family Support, Resilience, Affect Balance, and Perceived Stress

**Figure 2:**
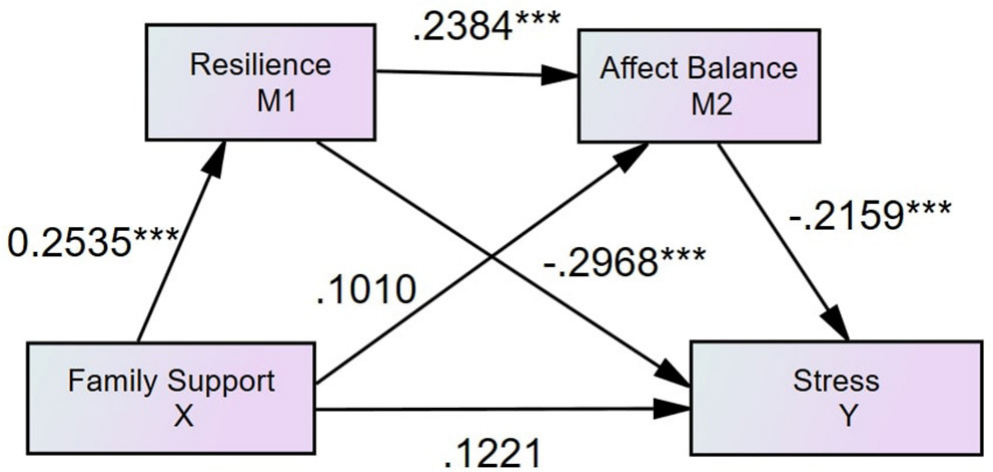
Standardized structural model (family support, *N* = 339, *R*^2^ = 0.2188). ***p < 0.001.

To evaluate the independent effects of family support, friend support, and specialist support were controlled as covariates in this serial mediation model. The results showed that resilience mediates the association between perceived family support and perceived stress (see Figure 2, Table 3). The direct path from family support to perceived stress (β = 0.1221, *p* = 0.3949) and the indirect path from family support to stress via affect balance was insignificant (β = −0.0259, 95% CI = [−0.0735, 0.0123]). The indirect path from family support to perceived stress via resilience (β = −0.0894, 95% CI = [−0.1778, −0.03626]) was significant.

**Table 3:** Bootstrapping indirect effects and 95% confidence intervals (CI) for the final mediation model.

**Model pathways**	**Point estimates**	**95%CI**	
		**Lower**	**Upper**
**Total indirect effect**			
Family support → Stress	−0.1308	−0.2183	−0.0588
Friend support → Stress	−0.0903	−0.1799	−0.0185
Specialist support → Stress	−0.2227	−0.3817	−0.1037
Perceived social support → Stress	−0.1064	−0.1650	−0.0595
**Indirect effect**			
Family support → Resilience → Stress	−0.0894	−0.1778	−0.0326
Family support → Resilience → Affect balance → Stress	−0.0155	−0.0335	−0.0043
Family support → Affect balance → Stress	−0.0259	−0.0672	0.0123
Friend support → Resilience → Stress	−0.0762	−0.1570	−0.0248
Friend support → Resilience → Affect balance → Stress	−0.0108	−0.0258	−0.0022
Friend support → Affect balance → Stress	−0.0033	−0.0453	0.0407
Specialist support → Resilience → Stress	−0.1525	−0.2917	−0.0570
Specialist support → Resilience → Affect balance → Stress	−0.0216	−0.0494	−0.0053
Specialist support → Affect balance → Stress	−0.0486	−0.1268	−0.0042
Perceived social support → Resilience → Stress	−0.0783	−0.1383	−0.0347
Perceived social support → Resilience → Affect balance → Stress	−0.0113	−0.0245	−0.0028
Perceived social support → Affect balance → Stress	−0.0169	−0.0312	−0.0044

#### Friend Support, Resilience, Affect Balance, and Perceived Stress

**Figure 3:**
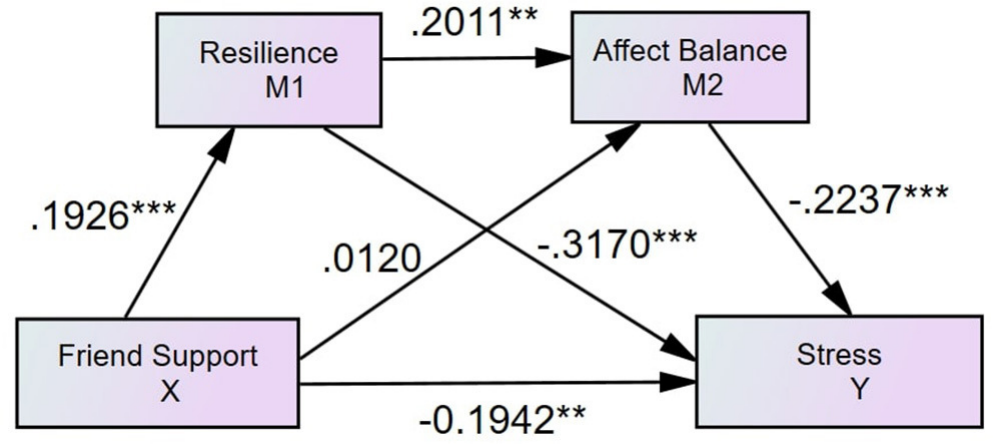
Standardized structural model (friend support, *N* = 339, *R*^2^ = 0.0820). ***p < 0.001, **p < 0.01.

After controlling family support and specialist support as the covariates, the results also demonstrated that resilience mediated the relationship between perceived friend support and stress (see Figure 3, Table 3). The direct effect of perceived friend support on stress was statistically significant (β = −0.1942, *p* = 0.0014). Moreover, the indirect path from friend support to stress via resilience was significant (β = −0.0762, 95% CI = [−0.1570, −0.0248]), but via affect balance (β = −0.0033, 95% CI = [−0.0453, 0.0407]) was insignificant.

#### Specialist Support, Resilience, Affect Balance, and Perceived Stress

**Figure 4:**
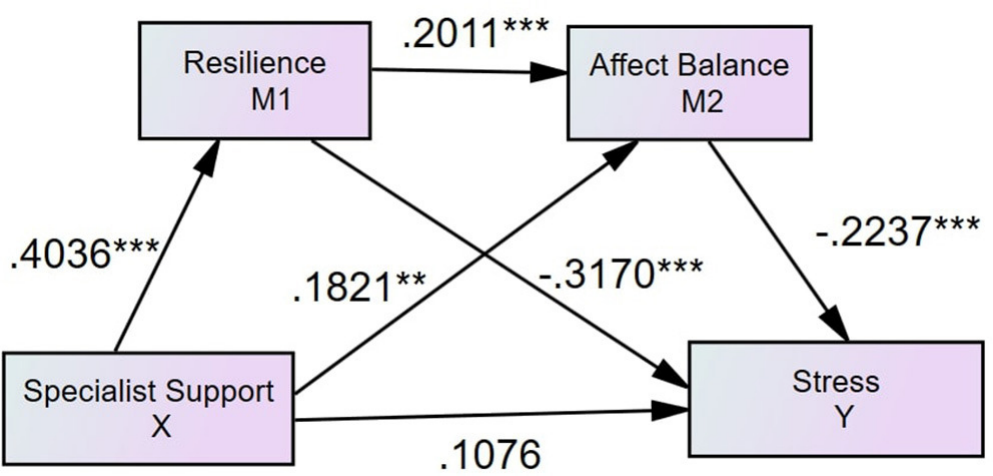
Standardized structural model (specialist support, *N* = 339, *R*^2^ = 0.2188). ***p < 0.001, **p < 0.01.

After controlling family support and friend support as the covariates, the results found that the association between specialist support and perceived stress is mediated by resilience and affect balance (see Figure 4, Table 3). The direct path from specialist support to stress was insignificant (β = 0.1076, *p* = 0.1873), and the path from specialist support to stress via resilience (β = −0.1525, 95% CI = [−0.2917, −0.0570]) and via affect balance (β = −0.0486, 95% CI = [−0.1268, −0.0042]) were significant.

### Serial Mediation

The results showed that the total indirect effects of family support (β = −0.0115, 95% CI = [−0.0232, −0.0007]), friend support (β = −0.0108, 95% CI = [−0.0258, −0.0022]), specialist support (β = −0.0216, 95% CI = [−0.0494, −0.0053]) on perceived stress through resilience and affect balance were significant (see Table 3).

## Discussion

The current study systematically analyzed the underlying relationship between specific perceived social supports (family, friend, and specialist, respectively), and perceived stress by examining the mediating effects of resilience and affect balance among SUD patients. It turned out that only perceived friend support has a direct effect on perceived stress among SUD patients. Moreover, resilience and affect balance fully mediate the associations between perceived family and specialist supports and perceived stress. In contrast, resilience partially mediates the association between perceived friend support and perceived stress. We found that the serial mediations, specific perceived social supports (friend, family, specialist) → resilience → affect balance → perceived stress, were significant in all models. The present study's findings implied that three types of perceived social supports, resilience, and affect balance are critical protective factors for SUD patients to reduce perceived stress.

Social support is generally considered as the effective caring from specific social networks (11). Family, friends, and specialist supports are three types of perceived social supports (36). The current study's findings showed that perceived social support's direct effects on perceived stress might vary by support types. Contrary to Hypothesis 1, the findings suggest that only perceived friend support directly affects perceived stress in SUD patients, which is inconsistent with the previous findings (48). One possible explanation of the insignificant direct effect of perceived family support on perceived stress is that the mediation effects of resilience and affect balance are strong enough to fully mediate the effect of perceived family support on perceived stress. Similarly, the explanation for the insignificant direct effect of perceived specialist support can also be attributed to the strong mediation effects of resilience and affect balance. However, the full mediations do not mean that perceived family support and perceived specialist support are not effective in reducing perceived stress among SUD patients. The *R*^2^ of family support and specialist support models are both 0.22 (see Figures 2, 4), suggesting that perceived family and specialist support exert significant effects on decreasing perceived stress through the mediators of resilience and affect balance that performs full mediation effects. Thus, it is recommended to design and develop support-based interventions for SUD patients. Future research is expected to explore how to design the relevant projects to boost family, friend and specialist support for SUD patients.

Consistent with Hypothesis 2, the findings suggested resilience mediates all the effects of three types of social supports on perceived stress among SUD patients, which aligns with the study conducted on Chinese soldiers (19). In particular, the findings suggest that resilience fully mediates the impacts of perceived family support and perceived specialist support on perceived stress in people with SUD. The findings were in line with the study conducted by Wu et al. (49) that the support provided by family members is a significant predictor of resilience. Moreover, the findings suggested that friend support can, directly and indirectly, reduce perceived stress (50). The support of peers can give SUD patients a sense of belonging and satisfy their need for recognition in society (51). Wolin and Wolin (52) also found that stable relationships with their peers and family members are conducive to the formation of resilience for SUD patients, enhancing confidence in coping with stress. The theoretical underpinning is that people who have adequate psychological capital and diversities of coping strategies are more likely to gain resilience, which is beneficial for reducing stress (53). Although the current research found that perceived friend support, directly and indirectly, affects perceived stress, it did not suggest that family support and specialist support are not as vital as friend support regarding reducing SUD patients' stress. Instead, the variances that family support model (*R*^2^ = 0.22, see Figure 2) and specialist support model (*R*^2^ = 0.22, see Figure 4) account for patients' stress reduction are higher than the variance that is explained by the friend model (*R*^2^ = 0.08, see Figure 3). Therefore, it indicated that rehabilitation centers are recommended to wisely and dynamically incorporate family, friend, and specialist supports during rehabilitation phases. Future studies may further investigate the effectiveness of interventions designed for developing family, friend and specialist supports.

Partially consistent with Hypothesis 3, the findings revealed that the associations between specialist support and perceived stress were mediated by affect balance. The findings corresponded with existing research conducted in other demographic groups (22). However, the paths from perceived family support and friend support to affect balance are insignificant. Based on the social learning theory (54), behaviors can be learned from social observation and interaction. As the study was conducted in compulsory rehabilitation centers, the detained individuals are obstructed to learn affect balance skills through the interaction processes with their families and friends. Overall, the findings implied that a higher level of support from specialist improves SUD patients' affect balance which in turn decrease their perceived stress.

The results further validate Hypothesis 4, which indicated that three serial mediation (family, friend, specialist supports → resilience → affect balance → perceived stress) were significant in all models. The findings indicated that resilience mediated the association between specific perceived social supports and affect balance. The findings also revealed that affect balance acted as a mediator of the association between resilience and perceived stress, which is in line with previous studies that SUD patients with a high perception of social support are prone to show more resilience (44). Precious studies suggest that those with higher resilience are more likely to experience higher affect balance (55), which in turn decreases perceived stress (56). The theoretical underpinning of the current findings is that different dimensions of perceived social support may promote resilience, while resilient individuals are good at balancing their affections, ultimately reducing stress perception.

## Limitations and Conclusions

However, this research also has some limitations that should be considered. First, the data of the study were collected from self-reported scales. Due to the limitations of self-report data, it is recommended to apply multiple assessment methods in future studies, such as collecting data from parents, communities, and rehabilitation center databases. Second, cross-sectional data implemented in this study cannot explore the causal relationship between variables. In further research, it is recommended to use mixed methods, such as longitudinal and experimental methods. Third, the current findings are only focused on the population of SUD patients with severe addiction level from compulsory rehabilitation centers, in which the findings may only apply to the individual with similar contexts. Future research is expected to have other groups, such as the SUD groups with mild and moderate addiction levels and non- SUD groups.

In summary, the current study aimed to examine the associations between three specific social supports (family support, friend support, specialist support), resilience, affect balance, and perceived stress among patients with SUD in China. The results showed that only friend support has a direct effect on perceived stress when resilience and affect balance are controlled. Secondly, we found that resilience played mediation roles in all models. Moreover, affect balance only mediates the effect of specialist support on perceived stress. These findings showed that family support, friend support, specialist support, resilience and affect balance are vital coping factors regarding reducing perceived stress among SUD patients.

## Data Availability Statement

The original contributions presented in the study are included in the article/supplementary material, further inquiries can be directed to the corresponding author/s.

## Ethics Statement

The studies have been approved by the Ethics Committee of Nanjing Medical University. The patients/participants provided their written informed consent to participate in this study.

## Author Contributions

CY, MX, TL, and YZ drafted the manuscript. CY contributed to data analysis, results and finalized the manuscript. MX contributed to introduction, method, discussion, and finalized the manuscript. TL revised made a significant contribution to manuscript revision. YZ revised and polished the manuscript. All authors have read and approved the final manuscript.

## Conflict of Interest

The authors declare that the research was conducted in the absence of any commercial or financial relationships that could be construed as a potential conflict of interest.
